# A qualitative study on the impact of death during COVID-19: Thoughts and feelings of Portuguese bereaved adults

**DOI:** 10.1371/journal.pone.0265284

**Published:** 2022-04-07

**Authors:** Ana Aguiar, Marta Pinto, Raquel Duarte

**Affiliations:** 1 EPIUnit—Instituto de Saúde Pública, Universidade do Porto, Porto, Portugal; 2 Laboratório para a Investigação Integrativa e Translacional em Saúde Populacional (ITR), Porto, Portugal; 3 Unidade de Investigação Clínica da ARS Norte, Porto, Portugal; 4 Faculdade de Psicologia e Ciências da Educação, Universidade do Porto, Porto, Portugal; 5 Instituto de Ciências Biomédicas Abel Salazar, Universidade do Porto, Porto, Portugal; 6 Serviço de Pneumologia, Centro Hospitalar de Vila Nova de Gaia/Espinho, Vila Nova de Gaia, Portugal; University of Milan, ITALY

## Abstract

As a global threat, the COVID-19 pandemic has been an important factor in increasing death rate worldwide. As the virus spreads across international borders, it causes severe illness, death, and disruptions in our daily lives. Death and dying rituals and customs aid bereaved people in overcoming their grief. In this sense, the purpose of this study was to access thoughts and feelings of Portuguese adults and the impact of the loss in daily life during COVID-19. A structured online questionnaire was applied (snowball sampling) and qualitative data on death and mourning namely the impact of the loss in daily life, was collected. One hundred and sixty-six individuals have lost someone since the beginning of the pandemic and were included. Analysis was inspired by Braun and Clark’s content analysis. Most participants were female (66.9%), the median age was of 37.3 years, and 70.5% had a high education degree. Moreover, 30.7% of the participants present anxiety symptoms and 10.2% depression symptoms. The answers of studied participants gave insights on the extent of the loss in day-to-day life and four thematic themes were found: (1) The perceived inadequacy of the funeral rituality, (2) Sadness, fear and loneliness, (3) Changes in sleeping and concentration and increased levels of anxiety and (4) Concerns regarding the pandemic situation. We found a high prevalence of anxiety and depression symptoms in the study sample. Also, the changes in post mortem procedures, have shown to be of great importance in the mourning procedure of the participants.

## Introduction

Every sector of the population is affected by the COVID-19 outbreak [1]. As a global threat, the pandemic has played a significant role in raising the global death rate. In the most recent years, other large-scale outbreaks have also caused disruptions, such as the Severe Acute Respiratory Syndrome (SARS), Avian influenza (H5N1), Middle East Respiratory Syndrome (MERS), and the Ebola virus disease epidemic [2]. The COVID-19 pandemic is the recent one on the list. As the new virus spreads across international borders, it causes severe illness, death, and disruption to our familiar daily lives [3].

In order to reduce the effects of COVID-19 on the population, safety measures such as social distancing and restrictions on visitations in healthcare institutions have been generally put into practice [4]. These changes can have harmful costs in the mental health [5] and should be carefully weighed. According to a published rapid review from the start of the pandemic, quarantine is frequently connected with a negative psychological effect [6]. Conferring to a recent qualitative study, people in healthcare settings reported difficulties interacting with personnel and receiving information about the patient’s condition, as well as worries about the quality of treatment offered [7].

All over the world, new guidelines and policies for the management of dead bodies, funerals, and burials are being implemented to contain the spread of infection [4, 8]. These guidelines could disrupt the bereavement process [7]. As Verdery et al. (2020) stated for one death related to COVID-19, an estimated number of nine persons were affected by the tragedy, demonstrating the widespread mourning and multiplying impact of loss [9].

Concerning death and rituals in the last moment, every group, community, or society has its own customs and rituals for death and mourning [10]. There are differences in such rituals and customs from one culture to another, however, they share a key ingredient that is the social connection [11]. Death and dying rituals and customs assist bereaved people in overcoming their grief [1]. These rituals and ceremonies apart from playing other roles like honoring the deceased, preparing him/her for acceptance in “the new world”, preserving the cultural legacy, and helping the bereaved to express their feelings [12], help in uniting people both with each other and with circumstances and collectives beyond themselves [13], offer a significant, culturally normative map for the expressions of emotions and help in adjusting in the environment without the deceased person [14].

The novel Coronavirus, on the other hand, transformed these treasured customs and rituals of death and dying all over the world [1]. These rites and ceremonies, which were once used to honor the departed and soothe the bereaved, have been curtailed or eliminated, and are now exclusively conducted by close relatives [8]. Reports of families deprived of the access to meet the patients dying from COVID-19; limited visitations to those suffering from other illnesses, denial to handover dead bodies, restrictions on funerals and other rituals, restrictions on number and relationship of mourners, and no close contact with the dead body are common [15]. During the COVID-19 pandemic, people suffer an unfinished mourning. First because those who follow the health protocols are prevented of conducting the rituals that are generally performed after death and, second, do not enjoy the company of others to help them cope with the pain produced by the loss [16].

Such restrictions are in direct conflict with the shared and symbolic reactions to an individual’s death. Recent studies have shown that attending funeral rites during COVID-19 pandemic provides better conditions for accepting grief and being able to cope with it, and that having the opportunity to participate in a mourning ritual, even for those who have died of COVID-19, can have a positive effect in assisting bereaved people to have a better mourning process [4, 16–18].

Grief is unavoidable and multidimensional for persons with losses [19]. However, the COVID-19 is making the mourning process more complicated whether or not the departed died due to COVID-19 infection or not. While dying alone may bring emotional anguish, mourning alone may be more difficult because the loss lingers, the connections change, and the space for reunion shrinks [20].

The lack of rituals and grieving, frequently results in disentrancement of grief and loss of social and cultural recognition that weakens support resources in assisting a positive grieving process. In this pandemic, more people are at risk of prolonged grief disorder (PGD) as a result of this strange, extended, and mourning alone event [19]. This does undoubtedly increase mental tension and a sense of guilt among mourners for grieving silently [1]. For this reason, with this qualitative work we aimed to access thoughts and feelings of Portuguese mourning individuals and the impact of the loss in daily life during COVID-19 pandemic.

## Methods

### Data collection

Data collection was performed through a structured online questionnaire, having as a base a snowball sampling method. The questionnaire was first posted via social networking platforms, such as Facebook, Instagram, LinkedIn, Twitter, and WhatsApp groups, from institutional (ISPUP) and personal accounts of the researchers, as stated elsewhere [21], in order to take advantage of the snowball sampling assumption. To each participant was presented a note at the end of the questionnaire asking to share the questionnaire with other 5 people.

Data was collected from November 10, 2020 until February 10, 2021 and the inclusion criteria were: be 18 years of age or older and be a resident in the country. A total of 929 participants accepted to participate in the study. From the total, 752 (80.9%) did not report any loss, 166 (17.9%) reported having lost someone during the pandemic and 11 (1.2%) did not want to answer. For the present study, participants who have lost someone since the beginning of the pandemic (n = 166) were considered. From these, a total of 85 provided information in the format of open-ended response.

The questionnaire included data on demographic characteristics, namely, and for the purpose of this study, we included gender, age and education level. Loss-related information included in the questionnaire complied information on the relationship to the deceased (i.e., partner, child, parent, grandparent, relative, friend, other relationship) and time since the loss in months were asked. To assess the impact of the loss an open-ended question was added to the questionnaire—“To what extent did your loss impact your day-to-day life?”–where participants could write freely about their loss. Furthermore, data on anxiety and depression symptoms were collected using the Hospital Anxiety and Depression Scale (HADS), Portuguese validated version [22]. The HADS scale is made up of 14 items, 7 of which are linked to anxiety symptoms and the other 7 which are related to depressive symptoms and to experiences experienced in the "previous week." Participants could choose from a total of four options in each question, which differed depending on the question. Each item is scored on a scale of zero to three, with three signifying the highest level of anxiety or depression. To classify the data for this investigation, we utilized the following cut off: a sum of zero to seven was defined as "normal," eight to ten as "borderline," and eleven to twenty-one as "case." When the HADS scale reaches 11, as proposed by Snaith, the presence of anxiety and depression symptoms is regarded indicative of "caseness" to a mood illness [23].

The anxiety and depression symptoms subscales had Cronbach alphas of 0.87 and 0.80, respectively.

### Ethical considerations

Ethical approval was obtained from the Ethics Committee of the Institute of Public Health of the University of Porto (CE20166). When accessing the online questionnaire, participants were first asked to give their informed consent by stating that they were 18 years of age or older and that they had read and understood the study goals. After, two options appeared: “I agree to participate in the study” or “I don’t agree to participate in the study”. If the first option was selected, informed consent was given and the questionnaire could start. Furthermore, no personal information that may be used to identify the participants was collected, and each was assigned a numerical ID.

### Data analysis

A qualitative research method was employed for this study. Qualitative research "(…) focuses on a phenomenological model in which reality is rooted in the subjects’ perceptions; the goal is to understand and find meanings through verbal narratives and observations rather than through numbers" [24]. Using Lloyd to complement the above statement, qualitative data provide answers to specific questions in the field of epidemiological studies, allowing for in-depth analysis of human behavior [25].

After a first reading of all answers, the protocol chosen for the analysis of the interviews was the protocol from Braun and Clarke (2006) designated thematic analysis [26]. The thematic analysis method allows the identification, analysis and report of patterns (themes) within the data. This allows the organization of the dataset in detail. The decision to chose thematic analysis is because it is especially beneficial for summarizing significant elements of large data sets since it forces the researcher to handle data in a well-structured manner, resulting in a clear and ordered final report [27].

In order to guarantee Trustworthiness, during thematic analysis, we followed the phases proposed by Nowell et al. (2017) [28]: 1) “Familiarizing with data” by read and re-read all quotations, taking notes concerning potential themes and by organizing the data in word processor in the means of a table; 2) “Generating initial codes” by involving a codebook created for this study; 3) “Searching for themes” in an inductive way (bottom up) since the themes named were strongly linked to the participants’ own discourses. Thus, this inductive (data driven) process is anchored in the coding of the data without forcing them to adjust to a pre-existing coding frame; 4) “Reviewing themes” as AA did the first coding process and MP reviewed and complemented the codes concerning the themes found in the responses until consensus was reached; “Defining and naming themes” in the most self-explicative manner. The final themes considered in the analysis were also reviewed and validated by RD as an additional member checking and production of the final codes and themes (Phase 6 “Producing the report”).

The design and findings of this qualitative study are reported following the consolidated criteria for reporting qualitative research (COREQ).

## Results

### Sample characteristics

A total of 166 participants have lost someone since the beginning of the pandemic. Most were female (66.9%), the median age was of 37.3 years, and 70.5% had a high education degree. Participants who had lost a grandparent consisted of 28.3% of the sample, father and/or mother 9.0%, and a friend 22.3% (Table 1). Moreover, 30.7% of the participants present anxiety symptoms and 10.2% depression symptoms.

**Table 1:** Characteristics of study participants, relative lost and prevalence of anxiety and depression symptoms.

*Variables*	n(%)
**Sex**	
Women	111 (66.9)
Men	54 (35.5)
Other	1 (0.6)
**Age (years) (mean (SD))**	37.3 (10.6)
**Education level**	
≤12 years	46 (27.7)
High education degree	117 (70.5)
Missing	3 (1.8)
**Relative loss**	
Grandmother	47 (28.3)
Mother/father	15 (9.0)
Friend	37 (22.3)
Other	61 (40.4)
**Anxiety symptoms**	
Non-anxiety	73 (44.0)
Moderate-anxiety	42 (25.3)
Anxiety	51 (30.7)
**Depressive symptoms**	
Non-depression	106 (63.9)
Moderate-depression	37 (22.3)
Depression	17 (10.2)

**Fig 1:**
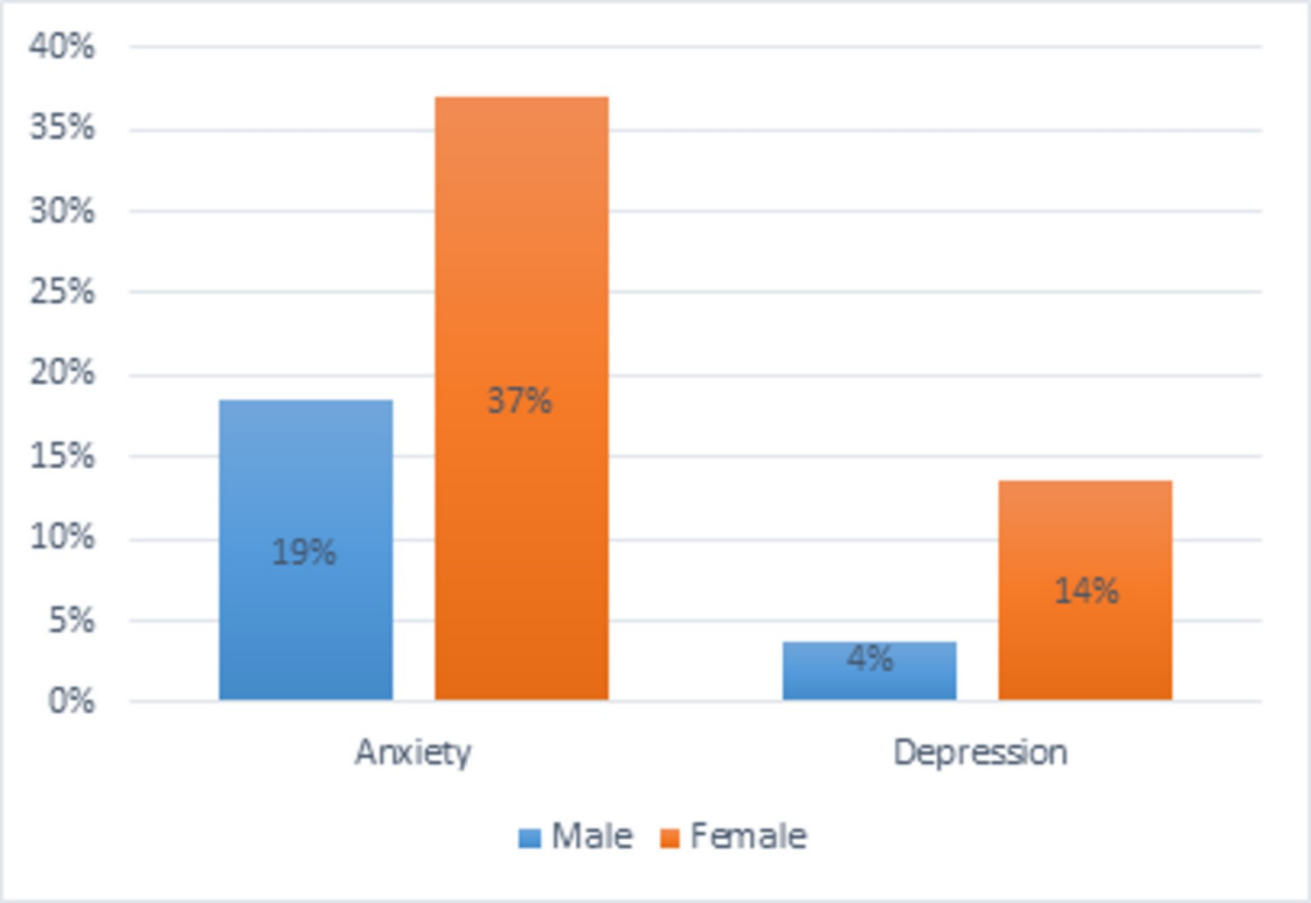
Anxiety and depression symptoms prevalence by gender.

As shown in Fig 1, the prevalence of both symptoms is higher in women comparing to men (anxiety symptoms is almost the double and the difference found is statistically different (p = 0.023) and depression is three times higher in women comparing to men although not statistically different (p = 0.061)).

### Qualitative analysis

The analysis of the open-ended questions (89 answers were given (53.6%)) identified four main themes: (1) The perceived inadequacy of the funeral rituality, (2) Sadness, fear and loneliness, (3) Changes in sleeping and concentration and increased levels of anxiety and (4) Concerns regarding the pandemic situation. Participant’s age is reported at the time of participation in the study. Below, we give an account of participants’ descriptions of the themes that have emerged.

#### (1) The perceived inadequacy of the funeral rituality

One of the most heinous aspects of COVID-19 is not only the fact that people are dying from it, but also the situations in which they die. Hospitals and healthcare facilities were closed to visitors due to self-isolation, prohibition of mass gatherings, and quarantine regulations. Many families are unable to say their final goodbyes to their loved ones, to attend burial rites, or to carry out their final grieving rituals. These difficult circumstances have upset not only certain family members, but also someone who is near death. The constant social and cultural removal of death, as well as its devastating effects during a period of high mortality salience such as the pandemic, led participants to recognize the critical psychological and social function funeral rites serve, as they help mourners feel embraced by their community and express their grief more fully. Friends and family members of people who died in hospitals with stringent visitation regulations struggled with the frustration of being unable to be present during the dying process. The bodies of individuals who died from COVID-19 could not be seen in person. Many participants expressed this:

*“Worse than the loss itself is the fact that we cannot mourn*, *we cannot attend ceremonies*, *we cannot comfort family and friends and receive consolation*, *we do not have the opportunity to be distracted in any way*, *the global news is slow to improve*.*”* (Female participant; 39 years; relative lost: aunt and father and mother from the best friend)

Funeral attendance has been severely limited after a person’s death. Family members must now host much smaller parties to choose which relatives, friends, and colleagues can attend in particular Christian traditions where a funeral is a celebration of the deceased’s life with loved ones. In practice, these formal requirements mean that mourners are unable to express physical comfort (through hugs or sitting next to each other during the funeral), are unable to touch the coffin, are unable to hold a reception after the funeral, and may not feel as if they have said the farewells they desired.

*“The fact that it was not possible to have a farewell with some “dignity” was what it cost more (…)”* (Male participant; 32 years; relative lost: grandmother)

Funerals help individuals acknowledge and accept the reality of death, share memories, convert their relationship with the person who died from one of presence to one of memory, give and receive social support, express their grief out loud, consider the meaning of life and death, and help start to think about how eventually to live life forward with meaning and purpose.

#### (2) Sadness, fear and loneliness

After the death of a loved one, many of the fears that run through our mind can be perceived as more of a threat than before the loss. If someone has witnessed someone die, the inevitability is more real than ever before in life [20]:

“*I face my mortality and the passage of time/ageing”* (Male participant; 49 years; relative lost: no blood relation)

When we have a fear-based thought about that person’s death, or about our mortality, or a worry about losing someone else, the body and mind are reacting stronger than before we experienced loss.

*“it made me think more about my life*. *Maybe I became more selfish*.*”* (Male participant; 41 years; relative lost: friend)

In grief, people can experience fear for many reasons. They can feel afraid of how the future has changed now that an important person in their lives is gone. People can feel frightened of more loss, worrying that we might lose more loved ones. Also, individuals can worry about their health, concerned that they may get sick or die soon, too. Some people get post-traumatic stress as a result of witnessing or having to hear details of the death.

*“Increased the feeling of fear of death”* (Female participant; 26 years; relative lost: uncle)

The intersection of grief and loneliness is also complicated. In the Encyclopedia of Mental Health (1998) researchers, Daniel Perlman and Letita Anne Peplau define loneliness as “The subjective psychological discomfort people experience when their network of social relationships is significantly deficient in either quality or quantity” as cited in Haley (2021) [29]. In other terms, loneliness happens when a person’s interpersonal wants or desires are not met by their social ties. Grieving people are at a disadvantage when it comes to loneliness since the person they long for has passed away.

“*Everything*. *The lack of everything*. *The loneliness”* (Female participant; 51 years; relative lost: husband)

Some losses, such as losing a spouse or a close companion, bring that overwhelming sense of loneliness. People live day by day with that person. They share so much of life that that person’s presence is absent in our life.

#### (3) Changes in sleeping and concentration and increased levels of anxiety

Death, understandably, causes people to become more anxious. Anxiety arises after a loss because losing someone you care about puts you in a vulnerable position [30]. Loss alters people’s lives on a daily basis. People are forced to confront their mortality as a result of it. And confronting these basic human facts about life’s unpredictability can bring fear and anxiety to the surface in unexpected and significant ways.

*“In different aspects*: *not sleeping*, *not being able to concentrate/think clearly*, *eating compulsively*, *frequent moments of anxiety*. *Greater impulsiveness and demotivation for all day-to-day tasks*. *Lack of empathy/patience*.*”* (Female participant; 49 years; relative lost: father)

Anxiety is a natural response of the attachment system to separation from a loved one.

#### (4) Concerns regarding the pandemic situation

The pandemic threat affects each individual in a particular way, according to one’s prior experiences, individual functioning aspects, contexts of life, protective resources, and perceived vulnerability. In the answers collected, we noted that participants revealed concerns regarding the pandemic situation in analogy with the lost:

*“Due to the infection conditions*, *it made me think about the ease and fragility that we have to be infected*. *We narrowly had close contact with the friend in question as well as my older elders*.*”* (Male participant; 36 years; relative lost: friend)

Also, the brief, temporary passage in life that each one of us has, is concerns reported.

*“It made me realize how ephemeral we are”* (Female participant; 26 years; relative lost: grandmother)

The COVID-19 situation is particularly stressful because it is hard to predict how things will develop, and our circumstances are changing rapidly. This can make us feel weak and as if we’ve lost control of our lives. We can’t control things in this situation, as we can’t control many things in our lives. These include the actions and reactions of other people, how long the situation will last, and what might happen in the future.

## Discussion

The lack of rituals and grieving, frequently results in disentrancement of grief and loss of social and cultural recognition that fades support resources in assisting a positive grieving course. The unusual process of grief in time of COVID-19 tests the usual process of coping with loss. For many years, the ‘five steps -denial, when they refuse to accept the diagnosis; anger or revolt; negotiation or bargaining; depression, mourning the loss of life, and eventually, acceptance of one´s finitude´ -were the usual framework used to analyse the psychological processing of loss. But with COVID-19, those practices were altered.

Physical separation and distancing during earlier outbreaks (such as the Ebola outbreak in West Africa) exacerbated feelings of grief, loss, misery, guilt, and helplessness among family members [31] and increase the risk for mental health problems. Mental health, being an integral part of the individual’s health, is defined by the WHO as “the state of well-being in which the individual acts with his own abilities, manages to deal with normal tensions of life, works productively and contributes effectively to the community” [32]. Despite being a clear definition, there are some features that should be emphasised. Namely the fact that, in mental health, there may be elements capable of generating mental disturbance without this meaning illness, since its development into mental illness is dependent on the interrelationship of various factors, internal or external, in which the individual face to certain risk factors may present disturbances or not [33].

To attempt to reduce infection rates and consequently mortality due to COVID-19, governments have implemented public health measures designed to reduce interactions between people. This includes limiting the number of mourners allowed at funerals and limiting interactions with the deceased during ceremonies, both of which effect all people who have lost loved ones during the current crisis [34]. This impossibility of farewell, of missing the interaction and care for the other, is indeed a problem that can cause further disruptions on the mental health status.

In addition to dealing with a traumatizing loss, because it happens in the context of acute disease, families are prevented from gradually preparing themselves emotionally. There was a high risk of contamination that impedes individuals to perform a proper funeral [26]. Consequently, in addition to the brutal loss of a loved one, people experience the impossibility to celebrate the final rites that give the opportunity of communion, complicity, connection with the sacred, and start a necessary process of detachment [27]. Paying one’s final respects to a loved one is a mental health gesture that allows people to make amends and go on with their lives [35]. Funerals were discontinued and required constraints on burials were imposed in the unusual regime created by the pandemic, causing more disruption than comfort [27]. In times of grief, people expect and rely on a degree of certainty regarding what will happen to their loved ones. During the COVID-19 pandemic, changes in mortuary practices have disrupted the usual and predictable treatment for the deceased [36].

Funerals rites are a well-documented means by which communities’ express emotion, say goodbye and heal. Mourning and funeral practices are being substantially reshaped due to COVID-19 [37]. Changing policies around burial and funeral rites may exacerbate feelings of uncertainty, loss and desperation, and new guidelines may affect the family’s ability to process loss as we were able to see on our study. Having no personal proof of death and not being able to bury a person in an acceptable way may make families and communities vulnerable to ‘ambiguous loss’ in which the experienced loss is not verified, the grieving process is frozen and the natural human need for meaning, sense, knowledge, connection and ritual is denied [38].

This void can have a continuing and devastating impact on everyday life, such as in work, and long-term mental health. As expressed by some of our participants, their losses have impacted in different ways by causing fear, sadness and the direct contact with the own death.

Concluding, altered grief and mourning and the difficult processes can disturb our capability to move forward and increase the risk of mental health complications such as depression and anxiety.

Strengths of the current study: 1) the study was conducted within a sample of a population-based setting; 2) a younger sample was recruited, which is different from the normal target audiences on qualitative research on grief; 3) the questionnaire was online and confidential which let participants answer openly and freely without the fear of being evaluated or judge. On the other hand, the limitations of the current study include: 1) almost half of the study sample didn’t answer to the open-ended question, which aimed to understand how the loss affect daily life of participants after the loss; 2) the findings cannot be generalized for the all population, since the recruitment was based on the principal of snowball sampling although that, having more them 80 open-ended answers is more than the usual seen in qualitative research; 3) since the data collection was made via online questionnaire and not in person by interview, more detailed information could not be accessed and analysed.
